# Implementation of an HPV vaccination program in Eldoret, Kenya: results from a qualitative assessment by key stakeholders

**DOI:** 10.1186/s12889-015-2219-y

**Published:** 2015-09-10

**Authors:** Heleen Vermandere, Violet Naanyu, Olivier Degomme, Kristien Michielsen

**Affiliations:** International Centre for Reproductive Health, Ghent University, De Pintelaan 185, 9000 Ghent, Belgium; Department of Behavioral Sciences, School of Medicine, College of Health Sciences, Moi University, Eldoret, Kenya

## Abstract

**Background:**

Cervical cancer strikes hard in low-resource regions yet primary prevention is still rare. Pilot projects have however showed that Human Papillomavirus (HPV) vaccination programs can attain high uptake. Nevertheless, a study accompanying a vaccination demonstration project in Eldoret, Kenya, revealed less encouraging outcomes: uptake during an initial phase targeting ten schools (i.e., 4000 eligible girls), was low and more schools had to be included to reach the proposed number of 3000 vaccinated girls. The previously conducted study also revealed that many mothers had not received promotional information which had to reach them through schools: teachers were sensitized by health staff and asked to invite students and parents for HPV vaccination in the referral hospital. In this qualitative study, we investigate factors that hampered promotion and vaccine uptake.

**Methods:**

Focus group discussions (FGD) with teachers (4) and fathers (3) were organized to assess awareness and attitudes towards the vaccination program, cervical cancer and the HPV vaccine, as well as a FGD with the vaccinators (1) to discuss the course of the program and potential improvements. Discussions were recorded, transcribed, translated, and analyzed using thematic analysis In addition, a meeting with the program coordinator was set up to reflect upon the program and the results of the FGD, and to formulate recommendations for future programs.

**Results:**

Cervical cancer was poorly understood by fathers and teachers and mainly linked with nonconforming sexual behavior and modern lifestyle. Few had heard about the vaccination opportunity: feeling uncomfortable to discuss cervical cancer and not considering it as important had hampered information flow. Teachers requested more support from health staff to address unexpected questions from parents. Non-uptake was also the result of distrust towards new vaccines. Schools entering the program in the second phase reacted faster: they were better organized, e.g., in terms of transport, while the community was already more familiarized with the vaccine.

**Conclusions:**

Close collaboration between teachers and health staff is crucial to obtain high HPV vaccine uptake among schoolgirls. Promotional messages should, besides providing correct information, tackle misbeliefs, address stigma and stress the priority to vaccinate all, regardless of lifestyle. Monitoring activities and continuous communication could allow for detection of rumors and unequal uptake in the community.

## Background

In Kenya, cervical cancer has the highest incidence and is the most lethal cancer among women, after breast and esophagus cancer. Yearly, almost 5000 women are diagnosed while close to 2500 die. East Africa is indeed one of the most affected regions of the world, with age standardized incidence and mortality four times as high as in more developed regions [1]. This health inequality gap is not only the result of limited screening and treatment options, also awareness of the disease and its symptoms is insufficient [2, 3]. If uptake of preventive measures remains inadequate, and taking into consideration the present population growth, the burden of cervical cancer could exponentially increase with over 50 % more new cases and deaths over the coming years [4].

Primary prevention through HPV vaccination has the potential to significantly reduce the incidence of cervical cancer and to eliminate cervical cancer disparity. The vaccines currently on the market are likely to prevent up to 70 % of cervical cancers, i.e., those caused by HPV 16 or 18 [5–7]. National vaccination programs are already rolled out in over 39 - mostly high-income - countries. In less wealthy regions, where the vaccines can have most impact, large scale vaccination efforts are still scarce [3, 4, 8]. Through demonstration projects, many low- and middle income countries have however gained experience regarding the introduction of the HPV vaccines. Results are very promising: high uptake is achieved (>70 %) and drop-out rates for second and third doses are low [9–14].

A longitudinal study linked with a demonstration program in Eldoret, Kenya, revealed however a different outcome: although the majority of mothers of eligible girls had expressed a wish to vaccinate their daughter before the start of the program (88 %; 253/287), only 31 % (79/254) of those who entered the follow-up study reported to have eventually done so. The main reason for non-uptake was lack of information on where and when the vaccination took place [15]. Poor promotion might thus have hampered the program. Other pilot programs already showed that thorough formative research followed by sensitization, especially through community influencers, is indeed key for success [11, 16, 17]. But as Kane et al. pointed out, one cannot expect similar results without investing a considerable amount of time and money to promotional activities [4]. Another reason for the noted difference in coverage might be a variation in the definition of uptake, and more particularly the targeted population (i.e., the denominator). Ladner et al. presented uptake rates of 21 demonstration projects; all achieved over 75 %. However, these figures might be overestimations for two reasons, as reported by the authors: 1) it is not clear what data was used in each study to calculate the target population which means the denominator might have been unreliable, and 2) programs might have targeted and recruited more girls than originally planned [10]. Post-vaccination studies could provide clarification. In the case of the program in Eldoret, it remains important to further investigate why coverage was insufficient and why people were ill-informed. By interviewing several stakeholders, further insight can be obtained and findings of the abovementioned study, in which women’s perspectives were assessed, can be triangulated. Including the male guardian, for example, helps clarifying whether women discuss cervical cancer prevention with their partner and whether they involve them in the decision (given that male partners often have decisive power [15, 18]). Also teachers are deemed important since they had an important task in this program, i.e., promotion of the vaccine, which was not well perceived by the women in the longitudinal study. Giving the teachers a voice enables us to understand how they experienced the program. Finally, the vaccinators themselves as well as the program coordinator can give insights regarding the organization and can reflect on the course of the program. The latter was in charge of promoting the HPV vaccination program among the teachers.

Many studies have already provided important insights from pilot vaccination projects. Through monitoring and evaluation, barriers are recognized, underserved populations are detected and effective sensitization and delivery strategies are identified [9, 11, 17, 19–25]. In general, internationally more attention is going to implementation research and process evaluations in order to improve and understand effectiveness of programs. Additionally, there is a call for a close follow-up of vaccination strategies: “Introducing new vaccines and ensuring they reach all people for whom they are intended is a challenging task, and the science related to implementing interventions effectively, efficiently, and with equity and high fidelity has received inadequate attention, particularly in African and Asian countries where overall research capacities are limited” [26].

In light of this, the aim of this study was to evaluate the implementation of the HPV vaccination demonstration program in Eldoret. In order to do so, three specific objectives were identified: 1) to verify whether fathers and teachers were aware about the program and had supported it, 2) to assess barriers in promotion, such as the level of understanding of cervical cancer and attitudes towards HPV vaccination, and 3) to gather recommendations, among fathers, teachers, vaccinators and the program coordinator, to contribute to the improvement of future HPV vaccination programs in Kenya.

## Methods

### The study context

The pilot HPV vaccination program - From May 2012 till March 2013, an HPV vaccination program was rolled out in Eldoret, Kenya. With support from the GARDASIL Access Program (GAP), Moi Referral and Teaching Hospital (MTRH) was able to vaccinate 3000 girls against cervical cancer. The vaccines were administered for free in the hospital on Wednesdays and Saturdays. Promotion took place in a pool of ten randomly selected schools as to avoid over-demand in the community. Through this, 4000 eligible girls, i.e., girls from class 4 to 8 (9–14 years old), were targeted. Health care providers went to the schools to inform the teachers who were then asked to promote the vaccine among the students and their parents. Each time, two pieces of information had to be passed: 1) basic information on cervical cancer and the HPV vaccine, and 2) practical information on the whereabouts of the vaccination program. However, due to poor response after three months, the program opened up to all other schools in the community, public and private.

Acceptance and uptake of the HPV vaccine – We assessed HPV vaccine acceptance among a randomly selected sample of women with eligible daughters in the ten initially included schools using a structured questionnaire (March 2012). During the interview, all women received basic information about cervical cancer and the upcoming HPV vaccination program. Once the program was completed, a follow-up survey was conducted to collect data regarding vaccine uptake (May 2013). Despite high baseline acceptance, reported uptake at follow-up was low. Main reasons for not receiving the vaccine were not feeling well-informed, fear of side effects and lack of time. In addition, women also reported that they had been confronted with opposition from people around them, among others their partner. More details about the program and the longitudinal study are described elsewhere [15].

### Recruitment of participants

The organization of the focus group discussion (FGD) was a stepwise process during which schools were randomly selected, asking the head teacher permission to set up a FGD in the school with either teachers or fathers. Each time a school was selected for a discussion, it was excluded from the pool (i.e., the ten schools that were targeted during the first wave of the vaccination program) to avoid two FGD in one school which could otherwise result in receiving the same information from both teachers and fathers regarding the organization of the program at school. Schools were invited until saturation was reached.

Once a school agreed to participate, the team set up the ideal date and time with a teacher, appointed by the head teacher, and participants were invited: 1) Fathers - Partners of the women who participated in the abovementioned longitudinal study were invited, hence they had a daughter who went to one of the targeted schools and had been eligible for vaccination. They were contacted by phone since contact information of the households had already been gathered during the cohort study [15]. 2) Teachers – The team invited all teachers present the day when permission to organize a FGD was asked. In addition, information letters were left behind inviting also other teachers to participate.

Recruitment of the vaccinators was done by contacting the head nurse responsible for the team, who then invited the other nurses for a FGD in the hospital. All FGD took place in May 2013. Finally, the program coordinator was directly invited by phone to meet in a place selected by him (October 2014).

### Procedures

All interviews were audio-recorded; FGD with fathers and teachers were moderated by researchers of the local team, who have considerable experience in conducting qualitative interviews regarding medical topics in the community. The discussion with the vaccinators and the validation meeting with the program coordinator were led by the first author of this paper. Before the start of the discussion, respondents were explained that participation was voluntary, that they could choose not to answer or leave the discussion at any point. Also signed consent forms were requested from all participants.

Interview guidelines for teachers and fathers were very similar and addressed awareness of the HPV vaccination program and whether or not they had participated in it. In addition, knowledge regarding cervical cancer and prevention was assessed, followed by a short, standardized informative session to provide correct information. The discussion ended by asking for recommendations on how future programs should be organized. During the FGD with teachers, extra attention was paid to their role as promoters and to their willingness to discuss cervical cancer prevention with their students.

The vaccinators were interviewed about two topics: their tasks during the program and whether they felt prepared, and how they thought the program could have been improved. Finally, a validation meeting with the program coordinator was organized to reflect on the vaccination program and the results of the FGD.

### Analysis

The interviews with the teachers and the vaccinators were in English, while the interviews with the fathers were in Swahili. English discussions were transcribed *verbatim* while Swahili sessions were translated and transcribed simultaneously, providing final transcripts in English. Transcription was done by local team members who at all-time could discuss interpretation of Swahili among each other. The transcripts were coded by two independent researchers, initially based on a list of codes deducted from the interview guidelines, focusing on awareness and perception of the HPV vaccination program, cervical cancer knowledge and attitudes towards the HPV vaccine. These codes were then gradually adapted and grouped into emerging themes [27]. Finally, results and conclusions of this analysis were discussed with the program coordinator to place them in the specific context of the HPV vaccination program and to formulate recommendations for future programs.

### Ethics statement

The study protocol was approved by the ethical boards of Moi University and Ghent University. All participants of FGD received a small compensation (200 KES, i.e., approximately 1.5€) to cover their time and transport cost. Written informed consent was obtained from all respondents.

## Results

### Characteristics of the participants

Seven schools were included given that saturation was reached after four FGD with teachers and three with fathers. In total, 67 teachers and fathers participated. FGD with teachers consisted of more female than male respondents and always included a mix of teachers of class 4 to 8, i.e., the classes targeted by the HPV vaccination program, and teachers of younger students. As there were no male vaccinators, the FGD with the nurses only included women (Table 1).

**Table 1:** Characteristics of participants of FGD

FGD	Participants	Number of participants			Teachers’ class		
#		Men	Women	Total	Class 1-3	Class 4-8^a^	Missing
1	Teachers of school 1	2	6	8	1	4	3
2	Teachers of school 2	2	10	12	-	9	3
3	Teachers of school 3	3	9	12	1	5	6
4	Teachers of school 4	4	7	11	1	3	6
5	Fathers of school 5	6	-	6	NA	NA	NA
6	Fathers of school 6	7	-	7	NA	NA	NA
7	Fathers of school 7	6	-	6	NA	NA	NA
8	Vaccinators (nurses of MTRH)	-	5	5	NA	NA	NA
	TOTAL	30	37	67			

^a^girls targeted for vaccination, approximately 9–14 years old

In total, 10 schools were enrolled in the first phase of the vaccination program. Later the program opened up to the entire community due to low uptake in the initial phase

FGD: focus group discussion; MTRH: Moi Teaching and Referral Hospital; NA: not applicable

### The HPV vaccination program

#### Knowledge about the program

Few fathers had heard about the past HPV vaccination program and when they had, it was mostly through their children and wives. When asked if they had discussed it with others, they explained that it was difficult given that it is taboo to openly discuss such topics. In addition, even if cervical cancer was brought up in conversations participants considered it a far-flung event, far removed from their own personal lives.

*Father (FGD 7): I heard of it from my children, that they are supposed to go and get checked.*

*Father (FGD 7): I only talked to my wife, not to other people. When talking about private parts to other people, they start drawing away from you.*

*Father (FGD 6): This is a new thing so we haven’t talked about it so much. Even if we hear about it, we don’t take it seriously….We’ve famous people like [name 1] being affected by cancer and went for treatment abroad….[name 2]….But to us it is a new thing.*

Similarly, not all teachers had received information about the HPV vaccination program or if the promotion had reached them, they “*took it lightly*” or “*didn’t pay so much attention*”. It was clear from all FGD with teachers that the health care providers had never sensitized the entire teacher corpse but rather a subset of teachers, appointed by the head teacher or those responsible for classes 4 to 8. As a result, in none of the schools an overall campaign or program was set up which led to misunderstandings and distrust.

*Teacher (FGD 4): They [the health care providers] met just some of the teachers, only those who were concerned with the… or those who had been given the duty of taking the children, because us we didn’t hear.*

A last reason why the program had not been discussed among participants was that cervical cancer, and health in general, concerns only women. Therefore, male teachers and fathers had not felt part of the vaccination activities.

*Teacher (male) (FGD 2): I think the name should be changed. You know, when I pass and I find a poster talking of cervical cancer. It bothers me less, I feel that I'm not of that part.*

*Teacher (female): To draw the attention of men.*

*Teacher (male): Men look at it and they see women’s issues.*

#### Participation in the program

Simply not being aware of the program or lack of information were the main reasons why not all teachers had cooperated in the program. Those teachers who had been involved, explained that they had just quickly passed practical information or invitation forms to their students, as opposed to also inform them about HPV and cervical cancer and encourage them to get vaccinated. Some teachers also remembered that parent meetings had been organized during which HPV vaccination was discussed.

*Teacher (FGD 2): I think that time we only mentioned. We were told [by health care providers] 'you tell these children to take their forms to the parents, those who are interested can go to MTRH for this'.... It was just as simple as that. We didn't think much about it.*

*Teacher (FGD 4): They [health care providers] came but there was a room which was organized for just the mothers, the parents of the girls who had accepted, so they talked to them and they went away.*

In terms of vaccination, some fathers and teachers reported having their girls vaccinated but most had not done so.

*Father (FGD 7): I took my children to all the three vaccinations.*

*Teacher (FGD 1): Yeah, I have heard about it, I even took my daughter.*

*Father (FGD 6): We didn’t take them.*

*Father (FGD 6): We didn’t know the importance of the vaccine but now we know.*

### Barriers of promotion: knowledge of cervical cancer and attitudes towards HPV vaccination

Given that the program was poorly known, we searched for reasons why promotion had failed and found two major reasons. First of all, due to a limited understanding of cervical cancer, prevention had not been considered a priority and many participants had not felt comfortable enough to discuss it. By providing correct information, participants did however welcome the HPV vaccine. Secondly, the new vaccine had instilled safety doubts which made people feel insecure to promote it.

#### Cervical cancer knowledge

Cervical cancer was poorly understood by fathers and teachers. For some of them, it was the first time they heard about it while other participants had problems with differentiating several types of cancer or distinguishing cervical cancer from other reproductive health conditions, such as fibroids or pelvic inflammatory disease.

*Father (FGD 7): I had heard about cancer but I didn’t know that there is cervical cancer. I always knew cancer is that which is caused by smoking. That’s what I knew.*

*Teacher (FGD 4): I think also when one is not clean maybe it can result into pelvic inflammatory disease, which can also lead to cancer, of the cervix.*

When asked about the causes of cervical cancer, many possibilities arose, yet HPV was rarely mentioned as a primary cause. Moreover, ‘cancer’ was interpreted in various ways: depending on the participants’ perception of causality, cervical cancer could be a wound, a rupture, an abnormal growth or swelling, a combination of diseases, an inflammation or an inherited condition. In turn, the ‘cancerous wound’ had many causal pathways, such as early sexual intercourse, coils (IUD), infectious diseases, (in)consistent use of contraceptives (pills or injection), unsafe abortion, accumulated dirt, rough sex and the use of sex toys.

*Father (FGD 5): I think when a girl engages in sex when young, if she develops a wound in the reproductive system and the wound takes long to heal, it might be the onset of cancer.*

*Father (FGD 5): Ok, I think a child is born while ‘fresh’ but when one becomes sexually active….in the process of coming into contact with several diseases especially the STIs…If the diseases are not treated, they block the reproductive organ which leads to something like cancer because I think cancer is nothing but a combination of several diseases.*

*Teacher (FGD 1): I think there might be, [a relation with bad hygiene] because if there is some dirt, let us say the accumulation, if it accumulates and accumulates and there is no attention taken to it or there is no cleanliness, that accumulation may stay there for long and it may cause, maybe, a wound and then from there a problem can develop.*

In general, participants either brought up risk factors related with sexual practices or with lifestyle. Sexual activities different from a monogamous, heterosexual relationship were mostly linked to cervical cancer. Examples of such practices are starting to be sexually active at young age, having sex during menstruation, having sex but not conceiving, masturbating (with dirty hands or objects), using and sharing sex toys, having multiple partners and having intercourse too soon after giving birth.

*Teacher (FGD 3): Also, when a mother is giving birth and then she gets an injury (pauses) and she goes for sexual intercourse before healing.*

*Teacher (FGD 2): We also have these habits that have cropped up nowadays. Eh, there is a practice of lesbianism and even sometimes they use sex toys. I don't know what standard of hygiene they reach to keep those things clean for them to share.*

*Teacher (FGD 3): Yes, I had a point… it is not only the machines they use. When you go to these children in boarding schools most of them use bananas and carrots.*

*Teacher: and the fingers*

*Teacher: and their fingers …They might be infected, they might be dirty.*

The majority of the fathers and teachers, yet not all, also thought ‘bad hygiene’ was potentially harmful, but this could be defined as either a lack of personal care, using dirty toilets or again engaging in certain sexual actions, such as masturbation or sex during menstruation.

*Teacher (FGD 1): When you have different sexual partners and you don’t pay attention to hygiene, you can get it.*

*Teacher (FGD 1): I think there might not be [a relation with bad hygiene] because I understand there are areas in Kenya where access to water is an issue and these people do not suffer from these diseases. But in urban areas, like here in Eldoret, in town so many people have such disease while these are the people who know how to wash, who know how to use even the vaginal soaps and still they are getting it.*

Furthermore, participants had different opinions whether or not cervical cancer was sexually transmitted. Similarly, heredity was also questioned by both fathers and teachers.

*Father (FGD 6): According to what my friend said that it is sexually transmitted, I don’t think it is true…. I had an aunt who was suffering from cervical cancer and died. The husband is still alive and he doesn’t seem to be having any problem.*

*Teacher (FGD 2): It is [inherited] because a new-born has directly inhaled everything from the parents. So even the blood of the parents who are cancerous, at least that kid would take some blood, which is cancerous.*

With regard to lifestyle, taking up ‘new or modern’ habits, whether it concerned smoking, food, cosmetics, medicines, contraceptives, using microwaves or exposure to X-rays, these behaviors were very often mentioned as ‘cancerogenic’. Especially contraceptives and food were of major concern, more particularly canned, packed or processed food or food exposed to fertilizers and chemicals. This resulted mostly out of the impression that cervical cancer, and cancer in general, is a disease of the rich, urban population. However, some teachers countered this and started to reflect on lack of diagnoses in remote areas. Likewise, one teacher questioned the relationship with contraceptives given that older women, who have never used such methods, are also affected.

*Father (FGD 6): A woman could plan with the man when to get a child but nowadays they use pills and injections. As days pass by they forget to go for the injection or to take the pills consistently. When this happens, they might cause a growth in the womb or they become toxic and cause cervical cancer.*

*Teacher (FGD 1): There are some older women who have suffered from cancer who have never used contraceptives, but their story is that they have had multiple partners, sexual partners earlier on when they were young, but they didn’t use contraceptives those days, it might not be, in my opinion it might not be a real reason.*

*Teacher (FGD 2): I’m just on the side of the food eaten by different people. People should …use indigenous food. Some of this food … The food colours, the chemicals they mix with this food. They facilitate different types of cancers. So people should turn to the indigenous food, the original African food.*

*Teacher (FGD 2): If you go in town, you’ll find that this is very common in town. As compared to the village and the remote areas. Why? Because, while in town, people eat different foods. Because of the living standards of the people, the living standard is high. People eat different food. Somebody can eat meat for six days in a week and a poor person can have meat maybe twice in a year.*

*Teacher (FGD 4): Many Africans are poor so most of us are dying, because of these things, so we are dying because of cervical cancer without knowing. Whenever we hear of it, it’s from those people who are able, maybe they go to London for checkups so you hear, ‘she was sick with cervical cancer’, and that is why we relate it to the rich. Otherwise we are dying without knowing it is the disease which is killing us.*

Finally, cervical cancer was perceived severe, affecting one’s fertility, and deadly; treatment was just too expensive. Preventive methods suggested by the participants were mostly abstinence of all aforementioned activities or products that could cause cancer. However, awareness was specified as the best prevention of all. One teacher even went further and mentioned that *“ignorance itself causes cancer”*.

*Father (FGD 6): When one hears the term cervical cancer especially when your child has it, you get scared. You then ask yourself whether she will ever give birth…Because when she has cervical cancer, she might not give birth and will finally die, so a parent loses hope.*

*Teacher(FGD 1): I have seen someone who suffers from it and was in a lot of pain and bleeding from the inside - where exactly, I don’t know, but somewhere in the uterus; it was very painful and was not curable.*

*Teacher (FGD 3): Ignorance of the ways of preventing cancer itself can produce cancer.*

*Moderator: can you give an example?*

*Teacher: When you use the gels [lubricants] for example, suppose they cause cancer…You see what has caused cancer is not the gel but ignorance.*

#### Attitudes towards HPV vaccination: drivers and barriers

Once participants were fully informed about cervical cancer and HPV vaccination, they were all accepting the vaccine. “*Prevention is better than cure*” was frequently brought up as main driver, together with the fact that the disease is deadly and cures are either unavailable or unaffordable. Fathers were especially in favor since it would protect their daughters’ fertility and therefore her future life as a mother. In general, foreseeing the girls’ future task such as providing grandchildren or taking care of the parents were reasons to consider HPV vaccination.

*Teacher (FGD 1): Now that you have taught us about it I think it’s good.*

*Father (FGD 6): If the father refuses to take his daughter - yet we are being told the disease can be prevented - he will be ruining his daughter’s life….she will not have children and may be unhappy in her marriage.*

Some teachers also pointed out a certain necessity for their pupils to be protected against cervical cancer. More particularly, they considered the students’ home situation or sexual activities as unsafe hence the need for prevention.

*Teacher (FGD 4): I can add, it is okay because we are living in a slum where the trend of prostitution is very high, and children are seeing those things going on and some of them are involved because of the status of their home, so I think it is ok.*

*Teacher (FGD 1): And I think it’s okay, and what should be done is that even our children should be taught, they should be sensitized, so if they are aware even this matter of having sex at an early age is not good because it gives rise to other diseases.*

Respondents also reported reasons why the vaccines could have been refused or why they themselves had not supported them. Several barriers concealed a certain level of distrust, towards vaccines in general or towards the HPV vaccine specifically. Bad experiences or rumors about other vaccines (polio and asthma especially) were brought up as to indicate the possible danger of vaccines, and the fact that this vaccine was new implied a potentially hidden experiment. Surprisingly, while protecting a girl’s fertility was a driver for accepting the vaccine, the same vaccine generated fear in terms of harming the girl’s fertility. In addition, several teachers thought that parents might have feared that vaccination would enhance sexual activity among the children.

*Father (FGD 6): There are parents who still have traditional beliefs and don’t believe in complementary medicine.*

*Teacher (FGD 4): Others think it is the disease of the rich (laughter); you know these chronic diseases, they think they are for the rich [after the moderator asked reasons to refuse the vaccine].*

*Teacher (FGD 1): We have not heard about people who have been vaccinated so we think they are starting with our children, they are used as guinea pigs or something, people try to see if it can work.*

*Father (FGD 6): There was a time we were told that when one is vaccinated, she might be unconscious for half an hour….I heard it somewhere and it prevented me from taking my daughter….*

*Father: Yes, I heard it somewhere. It scared me because I thought that was very dangerous.*

*Teacher (FGD 4): She [a mother of a student] was telling me that it is going to make our girls infertile, or maybe they will become sexually active, she said ‘me I refused my child to go for it’, but I didn’t ask anything more about it, so I left it at that. I was also of the belief that it has negative effects but now, I am for it.*

Finally, certain religious groups were known for rejecting all vaccines so participants mentioned them as refusers.

*Father (FGD 5): Religion is a very important factor. There are some religions which do not agree to treatment or vaccine.*

*Moderator: Which religion? Please give me examples.*

*Father: There is this church at our place with a red cross, Holy Spirit Church*

*Moderator: Yeah Holy Spirit Church*

*Father: Legion Maria and Wakorino*

During the short informative session and in the course of the remaining discussion, moderators often had to re-explain cancer-related issues or answer questions of participants. It was clear that once they had received the basic information, they started to interpret the obtained knowledge, each according to his or her capacity and according to his or her understanding of health and disease. For example, the fact that cervical cancer is sexually transmitted led to additional questions. Particularly male participants started wondering why boys were not targeted, given that they are “carriers of the virus”.

*Teacher (male) (FGD 4): Excuse me, somebody has talked about it being transferred from one woman to another by men, so men are carriers, I think also men should be vaccinated.*

Also eligibility was a topic of discussion. The moderator had to explain carefully that targeting young girls, in this case from class 4 to 8, was just a strategy to obtain girls who are not yet sexually active. Especially teachers were concerned about what would happen if a sexually active girl would receive the vaccine and whether or not they truly had to know which girls were already sexually active.

### Recommendations for future programs

Clearly, more information was requested by all participants, combined with facilitating HPV vaccination for parents, e.g. through school based vaccination. Furthermore, a stronger collaboration between health workers and teachers seemed essential for successful HPV vaccination.

#### Fathers and teachers

A first and very clear request from all participants was more sensitization, and any place or any channel would do: at churches, market places, schools, through radio, through community elders, etc. Everybody was welcome to help and spread information about cervical cancer vaccination but surprisingly, while churches were considered good venues, religious leaders themselves were not always seen as the correct source given that they have no medical background. Furthermore, fathers expressed the wish to be more included in health programs given that they considered themselves, often together with their spouse, the main decision taker regarding vaccination.

*Teacher (FGD 3): If people or ladies or girls or communities, if all people in general are taught about this cancer, let people know first about cancer and what brings cancer…Once they have the understanding of it, then they are going to take caution in the right way. But so many people don’t know about cancer. So let people learn about cancer, teach people about cancer! In schools, villages or where, wherever they can get the information….*

*Moderator (FGD 6): What about religious leaders, do they talk about it?*

*Father: Whenever they try we tell them they are not doctors.*

Secondly, the participants recommended to vaccinate at schools, as it would be more convenient for many parents. Moreover, some distrust towards hospitals or the health system in general was revealed which could be diminished by bringing the vaccines to the schools.

*Father (FGD 5): I heard about it [the vaccination program] but I lacked transport to take my daughter for the vaccination.*

*Teacher (FGD 3): Also going to the hospital will encourage bribing so we want to avoid that by taking it to school…because somebody tells you, bring something small so that I attend to you faster.*

*Teacher: And you might not even get the right vaccine even after giving out your bribes.*

Teachers were - “now that we are informed” - very keen to provide help and to promote the vaccines. They suggested themselves that they indeed should be the ones providing information given that they have day to day contact with the children. When asked, they claimed to feel comfortable to discuss such a topic in class, although some teachers showed some reluctance. For example, some of them would remind the others that in order to talk about it in class, it should be part of the curriculum, while others mentioned that it would be easier to discuss it with girls only. The latter statement was often rejected and led to discussions among participants regarding the importance to also inform boys. In the end, teachers did acknowledge that they wanted support by health workers to tackle difficult questions.

*Teacher (FGD 2): Teachers spent almost all their time with the children and children really listen to the teachers. Whatever teachers say, a child does not doubt. They can go home and convince the parent ‘this is what the teacher said’.*

*Teacher (FGD 4): I think what happens in a class, I think it should go hand in hand with the curriculum. I don’t see how this cervical cancer information can come, not unless it is also included in the curriculum.*

*Teacher (FGD 1): I have a different opinion. I think both the sexes should be told because nowadays they teach sciences about delivery, how the baby is formed and all that. I think they should teach in the same manner. So I think it is beneficial because they are growing. One time they will be parents and they need to have this knowledge.*

*Teacher (FGD 4): Or you can call a health worker to come and tell the parents.*

*Moderator: So you think it should be the health workers’ tasks?*

*Teacher: yes! Because I don’t have much experience. They might shoot questions that I don’t know how to answer, I may not be able to answer the questions.*

Thirdly, support from local authorities and the government was deemed essential, both in terms of assuring the safety and effectiveness of the preventive method as financially. Especially fathers were worried about the cost and thought the vaccines should be subsidized.

*Father (FGD 5): It shows I care about my daughters…and as I care, the government should do the same. It should be a national thing in schools and whatever. The vaccine should be taken to schools, to the ground.*

*Father (FGD 6): I agree with my colleagues because that amount is too high….the government should intervene because these children are our future leaders…He has talked of Kshs 2000 I would suggest Kshs 100 [referring to how much the vaccine should cost now that it was no longer available for free through the vaccination program] . With the current cost of living and if one has five children, it is a lot of money…One can try to get the 100 but 2000 [Kshs] is a lot of money.*

*Teacher (FGD 4): It is a good idea but I suggest, I think the government should do a bit of educating the masses because, if we teachers do not know what cervical cancer is, then how about that mother in the village, she will not accept; so education is very important.*

Finally, in all FGD people wanted to know when a next vaccination program would be organized, or where they could go to vaccinate their daughters given that now they were better informed, they did not want to wait any longer. Cervical cancer vaccination was now considered a priority.

#### Program coordinator and vaccinators

Similar to the teachers and fathers, the nurses stressed the need for information. More particularly, they stated that before the onset of the program they were unaware that cervical cancer is caused by a sexually transmittable virus. A short training before the start of the program, provided by the program coordinator, had informed them about HPV.

*Moderator (FGD 8): Before you were vaccinating the girls, were you aware that it was a sexually transmitted disease?*

*Nurse: Before that I didn’t know, until I was sensitized about that.*

In addition, the nurses also reported that they doubted their communication skills with the girls as to inform them about the vaccination, as well as how to address parents’ questions, e.g., why boys were not eligible. How to face these difficulties was not addressed in the training.

*Nurse (FGD 8): With the guardians, we were comfortable [discussing cervical cancer]. It is only that we thought with the children, of course they also have to know, but you could be wondering whether they understand, because someone who is like 9 years may not, in fact may not have started with reproductive or other health subjects. I was wondering if they understood, what we were talking about.*

While the program coordinator was surprised to hear that there were many teachers and parents unaware of the HPV vaccination program that had taken place, he offered some possible explanations based on his experiences. First of all, he had noticed that the attitude of the head teacher was crucial: during the program he saw that more pupils got vaccinated from schools with an enthusiastic and supportive head teacher. The nurses had perceived a similar effect. In addition, the coordinator confirmed that the health care providers visiting schools never spoke to the entire teacher corpse leaving it up to a few to further inform and involve their colleagues.

*Nurse (FGD 8): I think that it depended with how the authority of the school took this message. Did they take it with some weight, or did they just take it lightly…. So if they didn’t, then the girls would not appear. I think it depended on the authority of the school and how they received the message.*

Secondly, in one school teachers foresaw distance and thus transport time and cost as a major barrier for the parents, which made them doubt the feasibility of the program from the start. Lastly, during his contact moments with the schools he observed that two types of promotion were implemented: while in some schools the teachers informed the students who on their turn had to inform their parents, other schools organized contact moments with the parents to inform them directly. Likewise, some schools organized transport for the girls and a teacher accompanied them to the hospital to receive the vaccine. This was confirmed by the nurses.

*Nurse (FGD 8): Mostly they were brought by teachers, in groups.*

Especially schools that were not included in the first selection of 10 schools, tended to respond faster and more organized. These were often, yet not exclusively, private schools. The coordinator provided some possible explanations as to why these schools handled more swiftly: Private school teachers are considered more accountable for the well-being of their pupils, making it their responsibility to respond to vaccination efforts. Furthermore, both parents and teachers often have a higher socio-economic status compared with public school settings, making it easier for them to pick up and understand public health messages as well as to spend time and money for preventive medicine. Regarding the decision to open up the program to more schools, as opposed to, for example, revisiting the original selection, the coordinator explained that they called the ten schools to ask them to reinforce their promotional activities. However, teachers reported that parents were aware of the vaccination opportunity and were maybe simply refusing to vaccinate their daughters. As a result, the team decided to include more schools.

Finally, both the program coordinator and the nurses pointed out that the program knew a slow start but once it took off, demand increased exponentially. Particularly when the program opened up to more schools, the schools themselves started to inform neighboring schools inducing a type of snowball-effect.

*Nurse (FGD 8): At first, the message was not received kindly. Many people had questions, everyone had questions about this vaccine. So in the first place, I think it was considered like testing. Like someone wanted to know, ‘are others taking their children?’, but after that…most of them came and I think it was because they saw that almost everybody else was doing it.*

*Nurse (FGD 8): Yeah, in the beginning of the program, people were not willing, but towards the end, you see most of them are now coming and ask for the vaccine.*

## Discussion

The results clearly show that promotional activities were suboptimal: not all teachers were informed by health care providers, only some schools invited the parents for informative sessions (others relied entirely on students passing the invitation), and there were hardly any contact moments between health care providers and parents. Consequently, several bottlenecks were induced, blocking the flow of information from the health promoters, through the teachers and students, to the parents .

As stated before, target groups need to receive two pieces of information in order for them to undertake action to receive the HPV vaccine. First of all, they need to be aware of cervical cancer and they need to understand the importance of HPV vaccination. In order to achieve this, the information provided should correspond with the needs of the community. Secondly, potential participants need to know how they can receive the vaccine: where and when are vaccination activities rolled out?

Many women who participated in the longitudinal study, stated that none of this information had reached them [15], which was confirmed by the fathers in this study. However, the majority of the men also reported that their wife had informed them neither, meaning that many women had not shared the basic information they had received during the baseline interview. In addition, men are in general less informed given that they don’t feel addressed by public health campaigns regarding cervical cancer and that they find it particularly difficult to discuss it with others. Nevertheless, in case of an HPV vaccination opportunity they do want to discuss this with their wife and they do feel responsible for the final decision. Their lack of understanding might however result in vaccine refusal: opposition against the HPV vaccine by men was indeed reported as an important barrier by the women in the previous study [15]. Including men in cervical cancer prevention strategies and encouraging couples to discuss this might be challenging but seems crucial for success.

Following discussion will reflect first on the condition of awareness and understanding in the context of this demonstration project. Subsequently, the role of the teachers in public health programs will be discussed, more particularly to what extent teachers might take up certain types of promotional messages regarding HPV vaccination. Finally, the introduction of new vaccines will be assessed, i.e., how some people might need more time to gain confidence or to respond for them to adopt the new behavior, regardless of the information they received.

### Appropriate promotional messages

Besides the fact that many participants had not heard of the program, an equally important conclusion is that those who had received information had not given it thought and had not shared it with others. Fathers found it inappropriate to talk about cervical cancer with others while teachers stressed the need for more information for them to feel confident. However, there might have been other reasons. First of all, just like the fathers, some teachers felt equally uncomfortable to share this type of information with their colleagues or students. While they all wish to have a better understanding of cervical cancer, the topic causes them discomfort and anxiety.

Secondly, one might ask how participants, including the teachers, process and interpret the received information. How do they define viruses and transmission, what do they consider cancerogenic and who is at risk? During the discussions, it became clear that some had a very limited understanding of the human body and diseases. So even if the correct information was passed on, the question remains whether this newly gathered knowledge fitted into their vision of health and diseases and what they perceive as important to remember. For example, participants who knew about the cervical cancer vaccination program, still did not mention HPV as the main cause. Also in Vietnam and Italy, participants still had limited knowledge about cervical cancer after the implementation of an HPV vaccination program, even though they themselves considered them well-informed or had received the vaccine [20, 28].

Finally, and related with the previous argument, both teachers and fathers might not have perceived a cervical cancer prevention program as important: the strong conviction that cancer in general is a disease that affects rich people, or people with a “modern lifestyle”, provokes a certain indifference. Compared with a 2001 study from Gatune et al. (2005) in a rural area close to Nairobi, participants now stressed much more the causal relation with processed food or chemicals, rather than only sexual behavior and the use of contraception [29]. Given that participants did not feel part of this modern society exposed to those external, modern, risks, there was a strong overall feeling that cancer strikes others. Not observing cervical cancer among the general population is probably a result of lack of diagnoses and not discussing the sickness out of shame. The fact that participants did not perceive themselves or their environment susceptible for cervical cancer is however contradictive with previous findings where mothers reported that it was very likely that their daughter would have cervical cancer in the future [15]. The latter was of course a more direct and quantitative question concerning ones daughter which may have induced a socially desirable expression of concern while the FGD were more generally speaking.

Overall, we can conclude that translation of received information into action remains very challenging. Because of lack of understanding or not feeling addressed by promotional messages, people remain vulnerable for cervical cancer since they won’t feel urged to undertake actions to prevent it. Health messages should therefore go beyond providing essential information and should also address misunderstandings and rumors (e.g., *cervical cancer is not heritable and is not linked with the use of cosmetics*), assure that the target group is properly reached (e.g., *cervical cancer occurs both in urban and rural areas*), and actively fight stigma (e.g., *condom use can protect against cervical cancer* instead of *having multiple partners increases the risk of cervical cancer* or *cervical cancer is not caused by bad hygiene*). In order to identify the needs and worries of the target population, formative research should be carried out not only before the start of the program, but monitoring activities should continuously screen for new or evolving rumors [30]. Also, both men and women should be approached and empowered to discuss such a sensitive topic among each other. Moreover, support from the government and local authorities will increase the credibility of the program [25].

### Teachers as public health promoters

Besides receiving and sharing information, there were clearly other factors that influenced the HPV vaccination program. The program might have over-relied on teachers without considering their motivation or availability. Early involvement and clear communication with teachers regarding the design of the program was skipped, whereby taking up promotion could be more perceived as a favor towards the health staff instead of an agreement or responsibility.

However, even teachers who were addressed by health staff and had agreed on cooperation had not informed all their colleagues nor had they set up large-scale promotional activities. This failure to perform, may be caused by various factors. As in many low-income countries, Kenyan teachers might be poorly motivated due to little job satisfaction, few material tools, low salary, etc. [31]. Extra tasks might not be received well. Teachers requesting to include HPV and cervical cancer in the curriculum for them to discuss it in class, hints to the need for approval of the ministry of education as well as to delimiting work load. In addition*,* some teachers described their pupils’ background and behavior in a rather negative way, pointing out the worrying situation some students find themselves in. While this might be a driver for some teachers to help and protect the children, it might also pull some of them down.

Finally, talking about sexual health has always been a challenging task for teachers. Besides feeling uncomfortable to discuss such topics in class, some teachers might not agree with the type of information that should be shared or with what to promote (e.g., condom use vs. abstinence) [32]. Indeed, teachers often discussed whether or not boys should be informed as well, in which type of class cervical cancer could be discussed (is it the responsibility of the science teacher?), which age groups should be included, etc. Others even saw it as an opportunity to preach morality and discourage sexual freedom (i.e., masturbation or early sexual onset), using HPV and cervical cancer as a potential threat. Promoting it as a cancer vaccine and not mentioning the STI-aspect of cervical cancer was however never mentioned as an option. Teachers expected questions from both the students and the parents and therefore stressed the necessity to be well informed. It is also in this light that it becomes clear why teachers had only given their students the message to go to the hospital for vaccination as opposed to explaining them about HPV and cervical cancer: they opted to share logistical information rather than discussing prevention of a sexually transmittable disease.

So while school based vaccination was perceived as a good approach by all of the participants and while various studies have showed good results of such programs [33, 34], teachers should not stand alone when it comes to promotion. Health systems will have to support the schools, clearly describing and differentiating the responsibilities and messages that both parties will take up. As showed in a study by Brabin et al., close collaboration and good relationships between the schools and the health system are important predictors of vaccine uptake [35]. In addition, the schools might serve as a bridge between the health care providers and parents, whose contact is also crucial to achieve good coverage [34, 36]. Finally, the HPV vaccine might be seen as an opportunity to roll-out school health programs, including e.g., sexuality education, addressing the large but underserved group that are adolescents in low-income countries [37–39].

### Introducing new vaccines

New vaccines always provoke some hesitance and doubts, which diminish after a while but might linger for a very long time. These worries emerge from the fact that people have not yet seen the effects of the vaccine – or rather have not yet confirmed the absence of side-effects - but these concerns are also fed by persistent memories of bad experiences or rumors about other vaccines. Kennedy et al. showed that the combined MMR vaccine still causes worries in Scotland, after a controversy of more than 10 years ago, and even influenced decisions regarding new vaccines [40]. Likewise, participants in this study recalled stories of the polio vaccine and even an asthma vaccine, indicating previous failures of vaccine efforts and health communication. However, as reported by the vaccinators and the coordinator, the HPV vaccination program did eventually become successful, after a first period of habituation and trust gaining. Just like other new techniques, adoption of a vaccine might follow a Gaussian bell-curve of a normal distribution, representing diffusion throughout the community with early adopters setting the example while others lag behind (Diffusion of innovations, Everett Rogers). Indeed, people have reported a ‘wait and see’ approach when it comes to uptake of the HPV vaccine as to evade unknown side-effects [9, 41]. However, it will be important 1) to minimize the time span between adoption by innovators and laggards, and 2) to ensure that usage is not delayed among already underserved subpopulations, out-of-school youth or groups who refuse the vaccines for religious purpose. The high response noted during the second wave of this demonstration program may thus follow from late adopters coming round but might also reflect a difference between the ten selected schools and the newly included. Private vs. public schools was one of the aspects noted by the coordinator, indicating a potential threat for reaching health equity. Studies have indeed showed that ongoing HPV vaccination programs do not always eliminate cervical cancer disparity: girls from more deprived origins tend to have less chance to be fully vaccinated and non-school approaches may even induce more inequality [42–46]. Similar, parents with lower socio-economic background often have less cervical cancer knowledge and HPV vaccine awareness, which remains a first condition for uptake, while also financial restrictions impede vaccination [47–49].

In order to enhance acceptance and to speed up vaccine uptake, we need not only to spread information but we need to enter into dialogue with community members, addressing context specific concerns. What used to be predominantly a top-down approach, should become a continuous and open dialogue between all stakeholders [30]. In addition, surveillance programs should be put in place to assure that the HPV vaccines actually reach everybody – timely - and that they fulfill their potential to reduce the health inequality gap regarding cervical cancer.

### Limitations

The study has some limitations. First of all, selection of the vaccinators was not random, given that the nurses were invited by the head nurse. Although this might have induced selection bias, having duty around the time of the FGD was the major criteria for them to participate. In the end, five nurses participated in the FGD representing almost 50 % of the entire vaccinators team (i.e., twelve nurses).

Secondly, not all teachers who participated in the FGD gave classes to girls in class 4 to 8, i.e., the target group of the vaccination program. This means that these teachers were not asked to promote the vaccine among their students. Still, proper school based promotion would imply inclusion of the whole teacher corpse (maybe not in terms of active responsibilities but at least everybody should be informed). Moreover, their participation in the study revealed clearly that the vaccination opportunity was not discussed widely.

Thirdly, FGD with fathers were not transcribed *verbatim* in Swahili but were simultaneously translated into English. This may have led to the loss of some nuances or cultural specific concepts. In order to limit this type of error, researchers experienced in qualitative research in public health were given the task, while other local team members were always available to assist.

Finally, our study was conducted 14 months after the onset of the program which might have induced a recall bias. Participants had sometimes troubles remembering clearly what they had heard about the vaccination effort and from whom, or which promotional activities were organized. Especially the lack of insight in how promotion was implemented in each school limits the understanding on which channels were more successful than others. However, this also is a reflection of a lack of structural organization of, and exposure to sensitization.

## Conclusions

Although an HPV vaccination program had been implemented, people still had poor knowledge regarding cervical cancer. In general, cervical cancer prevention was not truly prioritized given that the disease is stigmatized through associations with non-accepted sexual activities and highly linked with usage of modern products such as cosmetics, contraception or processed food. Therefore many participants did not feel addressed by the promotion effort and had found it uncomfortable discussing the topic. Teachers pointed out that support from health staff would be essential in order for them to feel confident to promote the vaccine among students and parents. A closer collaboration with health care providers and schools would help to address questions of parents as well as teachers’ own doubts. Finally, distrust towards (new) vaccines had also hampered uptake: small-scale vaccination projects are often confused with trials, but also bad experiences during previous vaccination programs had reduced faith. Suspicion did however fade away after a couple of months, once the community was convinced about the safety of the vaccine. Also the inclusion of schools with higher capacities to respond to the vaccination invitation had boosted uptake.

Health care promoters of future programs will need to enter in dialogue with the community, as opposed to just provide information, to increase awareness and actively tackle misbeliefs and rumors. In addition, rolling-out HPV vaccination programs should go hand in hand with careful monitoring to assure that cervical cancer disparities are not further induced by differences in HPV vaccine coverage.

## References

[1] Estimated cancer incidence, mortality and prevalence worldwide in 2012. [http://globocan.iarc.fr/Pages/fact_sheets_population.aspx]

[2] Anorlu R (2008). Cervical cancer: the sub-Saharan African perspective. Reprod Health Matters.

[3] Steben M, Jeronimo J, Wittet S, Lamontagne DS, Ogilvie G, Jensen C, Smith J, Franceschi S (2012). Upgrading public health programs for human papillomavirus prevention and control is possible in low- and middle-income countries. Vaccine.

[4] Kane MA, Serrano B, de Sanjose S, Wittet S (2012). Implementation of human papillomavirus immunization in the developing world. Vaccine.

[5] Paavonen J, Naud P, Salmeron J, Wheeler CM, Chow SN, Apter D, Kitchener H, Castellsague X, Teixeira JC, Skinner SR (2009). Efficacy of human papillomavirus (HPV)-16/18 AS04-adjuvanted vaccine against cervical infection and precancer caused by oncogenic HPV types (PATRICIA): final analysis of a double-blind, randomised study in young women. Lancet.

[6] The Future II Study Group (2007). Quadrivalent vaccine against human papillomavirus to prevent high-grade cervical lesions. N Engl J Med.

[7] Li N, Franceschi S, Howell-Jones R, Snijders PJF, Clifford GM (2011). Human papillomavirus type distribution in 30,848 invasive cervical cancers worldwide: Variation by geographical region, histological type and year of publication. Int J Cancer.

[8] Markowitz LE, Tsu V, Deeks SL, Cubie H, Wang SA, Vicari AS, Brotherton JM (2012). Human papillomavirus vaccine introduction--the first five years. Vaccine.

[9] Ogembo JG, Manga S, Nulah K, Foglabenchi LH, Perlman S, Wamai RG, Welty T, Welty E, Tih P (2014). Achieving high uptake of human papillomavirus vaccine in Cameroon: lessons learned in overcoming challenges. Vaccine.

[10] Ladner J, Besson MH, Rodrigues M, Audureau E, Saba J (2014). Performance of 21 HPV vaccination programs implemented in low and middle-income countries, 2009–2013. BMC Public Health.

[11] LaMontagne DS, Barge S, Le NT, Mugisha E, Penny ME, Gandhi S, Janmohamed A, Kumakech E, Mosqueira NR, Nguyen NQ (2011). Human papillomavirus vaccine delivery strategies that achieved high coverage in low- and middle-income countries. Bull World Health Organ.

[12] Singh Y, Shah A, Singh M, Verma S, Shrestha BM, Vaidya P, Nakarmi RP, Shrestha SB (2010). Human papilloma virus vaccination in Nepal: an initial experience in Nepal. Asian Pac J Cancer Prev.

[13] Moodley I, Tathiah N, Mubaiwa V, Denny L (2013). High uptake of Gardasil vaccine among 9 - 12-year-old schoolgirls participating in an HPV vaccination demonstration project in KwaZulu-Natal, South Africa. S Afr Med J= Suid-Afrikaanse tydskrif vir geneeskunde.

[14] Watson-Jones D, Baisley K, Ponsiano R, Lemme F, Remes P, Ross D, Kapiga S, Mayaud P, de Sanjose S, Wight D (2012). Human papillomavirus vaccination in tanzanian schoolgirls: cluster-randomized trial comparing 2 vaccine-delivery strategies. J Infect Dis.

[15] Vermandere H, Naanyu V, Mabeya H, Vanden Broeck D, Michielsen K, Degomme O (2014). Determinants of acceptance and subsequent uptake of the HPV vaccine in a cohort in Eldoret, Kenya. PLoS One.

[16] Bingham A, Drake JK, LaMontagne DS. Sociocultural issues in the introduction of human papillomavirus vaccine in low-resource settings. Archives of pediatrics & adolescent medicine. 2009;163(5):455-461. 10.1001/archpediatrics.2009.50 19414692

[17] Galagan SR, Paul P, Menezes L, LaMontagne DS (2013). Influences on parental acceptance of HPV vaccination in demonstration projects in Uganda and Vietnam. Vaccine.

[18] Babirye JN, Rutebemberwa E, Kiguli J, Wamani H, Nuwaha F, Engebretsen IM (2011). More support for mothers: a qualitative study on factors affecting immunisation behaviour in Kampala, Uganda. BMC Public Health.

[19] Cover JK, Nghi NQ, Lamontagne DS, Huyen DT, Hien NT, Nga le T (2012). Acceptance patterns and decision-making for human papillomavirus vaccination among parents in Vietnam: an in-depth qualitative study post-vaccination. BMC Public Health.

[20] Paul P, Lamontagne DS, Le NT (2012). Knowledge of cervical cancer and HPV vaccine post- vaccination among mothers and daughters in Vietnam. Asian Pac J Cancer Prev.

[21] Ayissi CA, Wamai RG, Oduwo GO, Perlman S, Welty E, Welty T, Manga S, Ogembo JG (2012). Awareness, acceptability and uptake of human papilloma virus vaccine among Cameroonian school-attending female adolescents. J Community Health.

[22] Watson-Jones D, Tomlin K, Remes P, Baisley K, Ponsiano R, Soteli S, de Sanjose S, Changalucha J, Kapiga S, Hayes RJ (2012). Reasons for receiving or not receiving HPV vaccination in primary schoolgirls in Tanzania: a case control study. PLoS One.

[23] Wamai RG, Ayissi CA, Oduwo GO, Perlman S, Welty E, Manga S, Ogembo JG (2012). Assessing the effectiveness of a community-based sensitization strategy in creating awareness about HPV, cervical cancer and HPV vaccine among parents in North West Cameroon. J Community Health.

[24] Katz IT, Nkala B, Dietrich J, Wallace M, Bekker LG, Pollenz K, Bogart LM, Wright AA, Tsai AC, Bangsberg DR (2013). A qualitative analysis of factors influencing HPV vaccine uptake in Soweto, South Africa among adolescents and their caregivers. PLoS One.

[25] LaMontagne DS, Nghi NQ, Nga le T, Janmohamed A, Huyen DT, Hien NT, Tsu VD (2014). Qualitative study of the feasibility of HPV vaccine delivery to young adolescent girls in Vietnam: evidence from a government-implemented demonstration program. BMC Public Health.

[26] Arora NK, Lal AA, Hombach JM, Santos JI, Bhutta ZA, Sow SO, Greenwood B (2013). The need for targeted implementation research to improve coverage of basic vaccines and introduction of new vaccines. Vaccine.

[27] Braun V, Victoria C (2006). Using thematic analysis in psychology. Qual Res Psychol.

[28] Sopracordevole F, Cigolot F, Gardonio V, Di Giuseppe J, Boselli F, Ciavattini A. Teenagers’ knowledge about HPV infection and HPV vaccination in the first year of the public vaccination programme. European journal of clinical microbiology & infectious diseases : official publication of the European journal of clinical microbiology & infectious diseases. 2012;31(9):2319-2325. 10.1007/s10096-012-1571-4 22382817

[29] Gatune JW, Nyamongo IK. An ethnographic study of cervical cancer among women in rural Kenya: is there a folk causal model?. International Journal of Gynecological Cancer. 2005;15(6):1049-1059. 10.1111/j.1525-1438.2005.00261.x 16343181

[30] Larson HJ, Cooper LZ, Eskola J, Katz SL, Ratzan S (2011). Addressing the vaccine confidence gap. Lancet.

[31] Bennell P, Akyeampong K. Teacher Motivation in Sub-Saharan Africa and South Asia. 2007.

[32] Boonstra A (2011). Advancing sexuality education in developing countries: evidence and implications. Guttmacher Policy Review.

[33] Paul P, Fabio A (2014). Literature review of HPV vaccine delivery strategies: Considerations for school- and non-school based immunization program. Vaccine.

[34] Hopkins TG, Wood N (2013). Female human papillomavirus (HPV) vaccination: global uptake and the impact of attitudes. Vaccine.

[35] Brabin L, Stretch R, Roberts SA, Elton P, Baxter D, McCann R (2011). The school nurse, the school and HPV vaccination: a qualitative study of factors affecting HPV vaccine uptake. Vaccine.

[36] Hughes CC, Jones AL, Feemster KA, Fiks AG (2011). HPV vaccine decision making in pediatric primary care: a semi-structured interview study. BMC Pediatr.

[37] Chandra-Mouli V, Svanemyr J, Amin A, Fogstad H, Say L, Girard F, Temmerman M (2015). Twenty years after International Conference on Population and Development: where are we with adolescent sexual and reproductive health and rights?. J Adolesc Health.

[38] Gore FM, Bloem PJ, Patton GC, Ferguson J, Joseph V, Coffey C, Sawyer SM, Mathers CD (2011). Global burden of disease in young people aged 10–24 years: a systematic analysis. Lancet.

[39] Sexton C, Gerald L, Rager MK (2010). Annual high-quality wellness visits for adolescents: a standard whose time has come.

[40] Kennedy C, Gray Brunton C, Hogg R (2014). ‘Just that little bit of doubt’: Scottish parents’, teenage girls’ and health professionals’ views of the MMR, H1N1 and HPV vaccines. Int J Behav Med.

[41] Remes P, Selestine V, Changalucha J, Ross DA, Wight D, de Sanjose S, Kapiga S, Hayes RJ, Watson-Jones D (2012). A qualitative study of HPV vaccine acceptability among health workers, teachers, parents, female pupils, and religious leaders in northwest Tanzania. Vaccine.

[42] Jeudin P, Liveright E, Del Carmen MG, Perkins RB (2014). Race, ethnicity, and income factors impacting human papillomavirus vaccination rates. Clin Ther.

[43] Hughes A, Mesher D, White J, Soldan K. Coverage of the English national human papillomavirus (HPV) immunisation programme among 12 to 17 year-old females by area-level deprivation score, England, 2008 to 2011. Euro Surveill. 2014;19(2):1-6. 10.2807/1560-7917.es2014.19.2.20677 24457007

[44] Joseph NP, Clark JA, Bauchner H, Walsh JP, Mercilus G, Figaro J, Bibbo C, Perkins RB (2012). Knowledge, attitudes, and beliefs regarding HPV vaccination: ethnic and cultural differences between African-American and Haitian immigrant women. Womens Health Issues.

[45] Sacks RJ, Copas AJ, Wilkinson DM, Robinson AJ (2014). Uptake of the HPV vaccination programme in England: a cross-sectional survey of young women attending sexual health services. Sex Transm Infect.

[46] Spencer AM, Roberts SA, Brabin L, Patnick J, Verma A (2014). Sociodemographic factors predicting mother’s cervical screening and daughter’s HPV vaccination uptake. J Epidemiol Community Health.

[47] Gerend MA, Zapata C, Reyes E (2013). Predictors of human papillomavirus vaccination among daughters of low-income Latina mothers: the role of acculturation. J Adolesc Health.

[48] Sadry SA, De Souza LR, Yudin MH (2013). The impact of ethnicity on awareness and knowledge of and attitudes towards the human papillomavirus and vaccine among adult women. J Obstet Gynaecol Can.

[49] Reimer RA, Schommer JA, Houlihan AE, Gerrard M (2014). Ethnic and gender differences in HPV knowledge, awareness, and vaccine acceptability among White and Hispanic men and women. J Community Health.

